# Biological Characteristics and Medical Treatment of Breast Cancer in Young Women—A Featured Population: Results from the NORA Study

**DOI:** 10.4061/2011/534256

**Published:** 2010-10-04

**Authors:** P. Pronzato, G. Mustacchi, A. De Matteis, F. Di Costanzo, E. Rulli, I. Floriani, M. E. Cazzaniga

**Affiliations:** ^1^Oncologia Medica, IST, Genova 16010, Italy; ^2^Medical Oncology Department, University of Trieste, Trieste 34010, Italy; ^3^Medical Oncology C, National Cancer Institute, G. Pascale Foundation, Napoli 80010, Italy; ^4^Medical Oncology, Careggi University Hospital, Firenze 50012, Italy; ^5^Oncology Department, Mario Negri Institute for Pharmacological Research, Milano 20100, Italy; ^6^Medical Oncology, San Gerardo Hospital, Az Osp San Gerardo, Via Pergolesi 33, 20052 Monza, Italy

## Abstract

*Background*. The present paper described the biological characteristics and clinical behavior of young women in the cohort NORA study *Patients and Methods*. From 2000–2002, patients (*N* > 3500) were enrolled at 77 Italian hospitals. Women aged ≤50 years (*N* = 1013) were stratified into age groups (≤35, 36–40, 41–45, and 46–50 years). The relationship between age and patient characteristics, cancer presentation, and treatment was analyzed. *Results*. Younger women more frequently had tumors with ER/PgR-negative(*χ* ^2^ = 7.07; *P* = .008), HER2 amplification (*χ* ^2^ = 5.76; *P* = .01), and high (≥10%) Ki67 labelling index (*χ* ^2^ = 9.53; *P* = .002). Positive nodal status, large tumors, and elevated Ki67 all associated with the choice for chemotherapy followed by endocrine therapy in hormone receptor-positive patients (*P* < .0001). At univariate analysis, ER-ve status, chemotherapy and age resulted as the only statistically significant variables (HR = 2.02, *P* = .004, and >40 versus ≤40, *P* < .0001, resp.). At multivariate analysis, after adjustment for significant clinical and pathological factors, age remains a significant prognostic variable (HR = 0.93, *P* = .0021). *Conclusion*. This cohort study suggests that age *per sè* is an important prognostic factor. The restricted role of early diagnosis and the aggressive behavior of cancer in this population make necessary the application of targeted medical strategies crucial.

## 1. Introduction

The incidence of breast cancer increases with age but is not infrequent in women younger than age 40 years; approximately 2% of women are younger than 35 years when breast cancer is diagnosed [1]. Breast cancer has been reported to be more aggressive and to be associated with a more unfavorable prognosis in younger patients. Whereas management problems in older patients tend to be related to health and social aspects of the aging women, in young people, other factors, such as familial and reproductive problems, have to be considered. Trials of premenopausal or young breast cancer patients have been conducted or are ongoing, but data from clinical practice are lacking.

The principal aim of the NORA (National Oncological Research observatory on Adjuvant therapy in breast cancer) study was to describe treatment strategies and reasons for their selection in a population of breast cancer patients radically treated after surgery. This paper presents data concerning the biological characteristics of breast cancer and adjuvant treatment strategies in women ≤50 years, stratified according to age group.

## 2. Patients and Methods

The NORA survey was a multicenter, longitudinal, observational cohort study involving oncology centers at both academic and nonacademic institutions, which were distributed throughout Italy. Each center was required to register data on the first 10 consecutive patients treated in the years 2000, 2001, and 2002 (retrospective cohort) and on the first 20 consecutive patients who reached the oncology unit in 2003 (prospective cohort), for a total of 50 patients per center. These criteria were selected with the aim of maximizing enrollment while shortening the time needed to obtain an adequate followup period. Inclusion criteria included: first diagnosis of invasive breast cancer and absence of metastatic disease. Women affected by in situ carcinoma alone or who had undergone surgery with palliative intent (macroscopic residual disease) were considered ineligible. Concomitant participation in a clinical study did not qualify as an exclusion criterion as long as the proportion of these patients remained below 20% and 40% in the retrospective and prospective cohorts, respectively. Patients enrolled in a clinical trial who exceeded these rates were not considered for the analysis, but only registered. Data on demographic characteristics, familial and pathological history, diagnostic methods, surgery, pathological features, and adjuvant treatments were collected. In order to collect data on changes in adjuvant treatment, toxicity, and cancer-related events, all patients were followed up every 6 months for a minimum of 4 to a maximum of 8 years.

The study complied with the requirements of Italian law regarding observational studies. The nature and purposes of the survey were explained in detail to all potential participants, and their consent to data handling according to Italian privacy regulations was obtained. Assuming involvement of approximately 70 centers with a minimum recruitment of 50 patients, investigators planned to enroll approximately 3500 women. This number allows an estimate of the distribution of adjuvant strategies with a 95% confidence interval (CI) range of no more than 3%.

This paper presents data on the biological characteristics and medical treatment of women ≤50 years old. To better study the influence of age on patterns of care and the distribution of selected factors related to patient characteristics, we identified four groups: ≤35, 36–40, 41–45, and 46–50 years. The characteristics of selected factors across age groups were described by relative and absolute frequencies. We were not able to detail menopausal status, mainly because data in the retrospective cohort were lacking or largely incomplete. Analyses were performed using the Mantel-Haenszel test for trend and the chi-square test for heterogeneity. Cox's model was applied for univariate analysis. Unless otherwise specified, all tests were within one degree of freedom.

## 3. Results

A total of 3532 breast cancer patients were enrolled by 71 Italian centers. Seventeen patients (0.5%) were subsequently excluded because of synchronous tumors, leaving 3515 evaluable patients. Academic institutions comprised 21.2% of the centers; 42.3% were located in northern Italy, 28.2% in central Italy, and 29.6% in southern Italy and the islands. Therefore, the institutions were well distributed and representative of the country.

Baseline characteristics and pathological features of the entire patient population have been reported previously [2]. Briefly, the median age of the patients in the whole population was 58 years (range 25–92); almost all of them had a good ECOG PS (0-1: 98.2%) and were postmenopausal when breast cancer was diagnosed (72.3%). Approximately one-third of the patients had a positive history of cardiovascular (24.1%) or gynaecological (13.2%) comorbidities. At the time of study entry, 1013 (28.8%) patients were ≤50 years (Table 1), and most of them were not menopausal (79.8%). The median age was 44.4 years (minimum-maximum value: 24–50). Comorbidities are infrequent in the majority of the patients <35 years (18.2%) and tend to be more present with increasing age (*χ* ^2^ = 16.87; *P* < .0001), particularly skeletal diseases.

Breast cancer was detected by self-examination in the majority of patients ≤35 years (80%) and by periodic screening programs in those 40–45 years (23.5%) and 45–50 years (23.8%), which is similar to that observed in the whole population (Table 2).

Conservative surgery was performed in 66.4% of the cases, without any significant difference across age groups (*χ* ^2^ = 1.35; *P* = .24). In the ≤35 age group, mastectomy was performed in 34.6% of the cases, despite the fact that small tumors (<2 cm) were found in 57.7% of the patients. Sentinel node technique was used together with conservative surgery in 14.1% of the cases, without any difference according to age group (*χ* ^2^ = 0.02; *P* = .87). 

Histologic characteristics were similar among all subgroups, except for the lobular subtype, which tended to be more frequent among women older than 35 years and was rare in women ≤35 years (10.5% versus 2.6%, resp.). 

### 3.1. pTN Stage and Biological Characteristics

T stage and nodal involvement (TN) as well as the chief biological characteristics are reported in Table 3. The distribution of TN stage did not differ according to age group (*χ* ^2^ = 2.18; *P* = .13); on the contrary, hormone receptor status, human epidermal growth factor receptor 2 (HER2) amplification, and elevated Ki67 labelling index showed statistically significant correlations with age (Tables 4 and 5), being more frequently present in the group younger than 35 years. Thus, the tumours of younger women were more frequently estrogen receptor (ER)/progesterone receptor (PgR)-negative (*χ* ^2^ = 7.07; *P* = .008), HER2 amplified (*χ* ^2^ = 5.76; *P* = .01), and with high (≥10%) Ki67 labelling index (*χ* ^2^ = 9.53; *P* = .002).

### 3.2. Adjuvant Medical Treatment

The choice of adjuvant medical treatment also correlated with age. Endocrine therapy alone was more frequently administered to women older than 35 years (≤35: 0%; 36–40: 7.4%; 41–45: 14.9%; 45–50: 20.1%) whereas younger women were more likely to receive chemotherapy alone or chemotherapy followed by endocrine therapy (*χ* ^2^ = 35.43; *P* < .0001). If we analyse the choice of adjuvant therapy according to the hormone status (Table 6), we observe that almost all patients who were ER+/PgR+ received endocrine therapy alone or chemo- followed by endocrine therapy, independently of age, even if older patients were most likely to be treated with a treatment containing hormones, in comparison to younger ones. This finding is consistent with the more frequent presence of hormone receptors in older women (*χ* ^2^ = 699.89; *P* < .0001). 

No differences were observed in the type of adjuvant chemotherapy (*χ* ^2^ = 12.09; *P* = .2). Anthracycline-based chemotherapy was the preferred choice in the whole group (65.2%) as well as in all subgroups of patients (Table 7).

Endocrine therapy alone was administered in 151 patients. Eighty-one patients received tamoxifen and 70 a luteinizing hormone-releasing hormone (LHRH) analogue with or without tamoxifen. Of 636 patients who received chemotherapy followed by endocrine therapy, an anthracycline-based regimen was chosen in 352 (55.8%); cyclophosphamide, methotrexate, and fluorouracil in 233 (37%), and a taxane-based therapy in 39 (6.2%).

The choice between endocrine therapy and chemotherapy was mainly based on the patient's hormone receptor status. Endocrine therapy was widely used in hormone receptor-positive patients, while chemotherapy was preferred in patients who were hormone receptor-negative (*χ* ^2^ = 699.89; *P* < .0001). 

In the hormone receptor-positive population, the preference for chemotherapy followed by endocrine therapy was strongly influenced by nodal status (N+ versus N−: 69.6% versus 97.9%; *χ* ^2^ = 102.20*; P* < .0001), T stage (T_1_ versus T>1: 73.8% versus 95%; *χ* ^2^ = 56.15*; P* < .0001), and elevated Ki67 (≤10% versus >10%; *χ* ^2^ = 15.43*; P* < .0001). The lack of expression of PgR did not seem to influence the choice between endocrine therapy alone or chemotherapy followed by endocrine therapy (*P* = .6), but no conclusion could be drawn regarding the influence of HER2 status because of the high percentage of missing values. The menopausal status did not have any relevance on the choice between chemotherapy or endocrine therapy (*χ* ^2^ = 0.68; *P* = .71).

**Figure 1:**
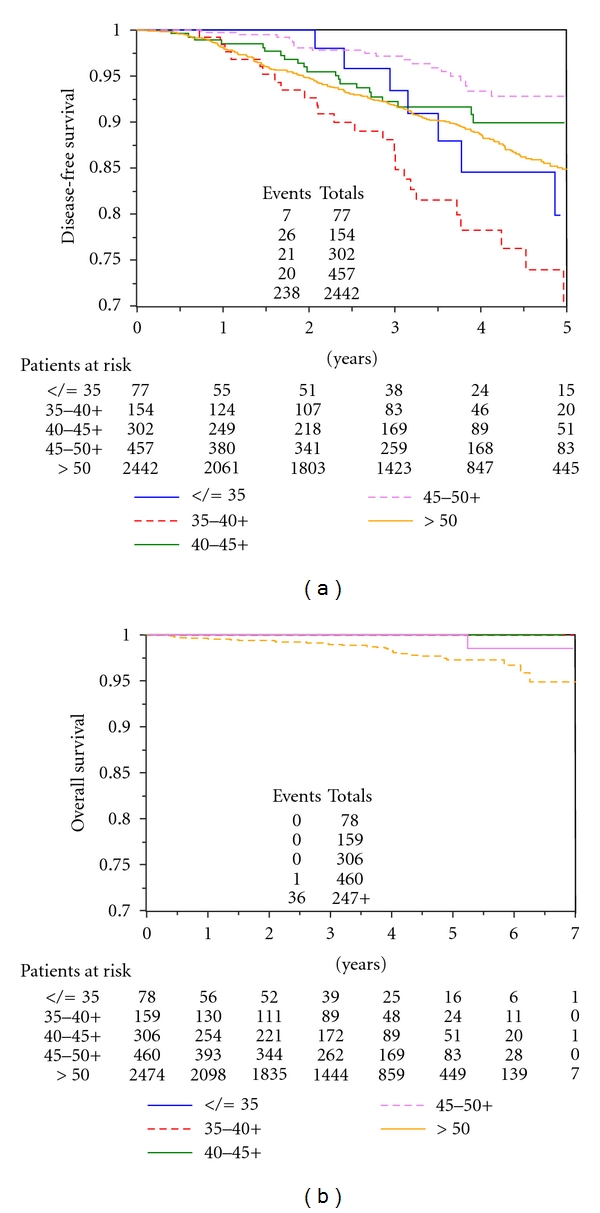
(a) Disease-free survival (DFS), and (b) overall survival (OS) according to age group.

There was a statistically significant difference among age groups in terms of DFS (Figure 1(a)). The worse prognosis was observed in those aged 35–40 (*χ* ^2^ = 27.69*; P* < .0001). There were no significant differences between age groups in terms of overall survival (OS); however, OS was statistically better in younger patients compared with the total population studied (Figure 1(b)).

At univariate analysis, ER-ve status (HR = 2.02, *P* = .004), choice for chemotherapy (HR = 4.45, *P* = .019), and age (<40 versus ≤40, HR = 0.34, *P* < .0001) were independent variables all associated with worse DFS.

At multivariate analysis, when adjusted for the clinical and biological factors significant at univariate analysis, age remains a statistically significant independent variable (HR = 0.39, *P* < .0001) for worse DFS.

## 4. Discussion

The population analyzed in this paper consisted of 1014 women ≤50 years of age. As expected, the majority of the patients did not have the comorbidities observed in older women [3] that can limit the choice of treatment. Yet even among women aged 41–50, the risk of cardiovascular, skeletal, and gynecological disease may be important enough to have an impact on treatment selection. 

At the time patients entered the cohort study, evidence did not support a screening mammogram in these women. Nevertheless, 34.3% of the patients in this age group had their breast cancer diagnosed by a periodic screening test. This could be interpreted as a sporadic finding. Debate continues about the validity of a screening mammography program for women younger than 45 years. Issues include the lower sensitivity of mammography in this subset of patients, the high rate of false-positives, and health care costs. However, the high rate of women spontaneously screened in this study could be the starting point for a more rational and cost-effective approach in this age group.

 These data demonstrate that tumors that occur in very young (<35 years) women are characterized by a lack of hormone receptors and a high proliferation rate. Our results are similar to those described by Colleoni et al. [4]. This study was a cohort trial; thus no centralized revision of HR status or Ki67 was planned; concerning the HER2 status, we must keep in mind that these data were collected starting from 2000, when the determination was not routinely applied. With HER2-neu status being determined more often, very young women with breast cancer are more often found to have tumors that amplify HER2-neu in comparison with older patients (*χ* ^2^ = 5.76; *P* = .01).

In young patients, appropriate therapies may ameliorate the unfavorable prognostic features associated with age. In a study by Kroman et al. [5], young patients who did not receive adjuvant therapy had a worse prognosis than older patients with the same biological characteristics whereas for patients who received adjuvant treatment, irrespective of age, a worse prognosis was not evident. 

While chemotherapy was administered to almost all women who were ER/PgR−, and endocrine therapy was given to patients with tumors that were clearly endocrine-responsive, the sequence of chemotherapy and endocrine therapy was chosen for the majority of patients with tumor expression of at least one of the two receptors. This treatment choice in patients with endocrine-sensitive breast cancer has been vigorously discussed. Some retrospective analyses have argued that the value of such a treatment sequence is limited in patients with endocrine-positive disease [6]. However, the INT 0101 trial [7], which randomized premenopausal patients to chemotherapy alone, chemotherapy plus LHRH analogs for 5 years or the same treatment with the addition of tamoxifen, demonstrated the superiority of the latter arm over the other two choices. 

These results are similar to those described by Regan et al. [8], who recently analyzed the determinants for choosing chemotherapy in a population of hormone receptor-positive premenopausal patients enrolled in three different International Breast Cancer Study Group trials. Positive nodal status, higher grade tumors, and large tumors were all factors for choosing chemotherapy.

In premenopausal women, the effectiveness of chemotherapy may also be related to the induction of menopause, which has an endocrine-like effect. Studies comparing endocrine therapy with chemotherapy have not demonstrated relevant differences [9–11]. However, with the development of second- and third-generation chemotherapy regimens such as CEF, dose-dense paclitaxel, and dose-dense EC Taxol, which are more effective than standard Bonadonna CMF, and AC alone, no final conclusions should be drawn from studies that compare “first generation” chemotherapies with ovarian ablation. In this cohort study, it was quite clear that Italian oncologists preferred the use of sequential chemotherapy and endocrine therapy. However, in most cases, the choice was for a “new” anthracycline-based regimen.

## 5. Conclusions

In young patients with breast cancer, management problems specific to this patient population could arise. In this cohort study, investigators collected data on a large series of patients aged ≤50 years who were treated in clinical practice throughout Italy, outside of controlled clinical trials. The results confirm previous findings obtained by other observational trials, in particular, evidence of unfavorable biological and pathological patterns. The concept of young age as a negative prognostic factor most likely led oncologists to choose more aggressive treatments, such as chemotherapy. Because of the restricted role of early diagnosis and the aggressive behaviour of cancer in this patient population, the application of validated medical strategies is of critical importance.

## Figures and Tables

**Table 1:** Age distribution at the time of study entry.

Age (years)	*N*	%
≤35	78	7.7
35–40	163	16.1
41–45	308	30.4
46–50	463	45.8

Median age: 44.4 (minimum-maximum: 24–50).

**Table 2:** Diagnosis of breast cancer—*n* (%).

	≤35 (%)	36–40 (%)	41–45 (%)	46–50(%)	Total (%)
Self-breast examination	56 (80.0)	92 (66.7)	151 (56.3)	194 (49.6)	493 (56.9)
Periodical screening	3 (4.3)	9 (66.7)	63 (23.5)	93 (23.8)	168 (19.4)
Occasional finding	11 (15.7)	37 (26.8)	53 (19.8)	101 (25.8)	202 (23.3)
Other					4
Missing					145

*χ* ^2^ = 45.53; *P* < .001.

**Table 3:** TN stage according to age groups—*n* (%).

	≤35 (%)	36–40 (%)	41–45 (%)	46–50(%)	Total (%)
*P*T_1_*	45 (57.7)	88 (54.7)	194 (63.0)	294 (63.8)	621 (61.6)
*P*T_2_	30 (38.5)	63 (39.1)	103 (33.4)	147 (31.9)	343 (34.0)
*P*T_3_	2 (2.6)	10 (6.2)	5 (1.6)	12 (2.6)	29 (2.9)
*P*T_4_	1 (1.3)	0 (0.0)	6 (2.0)	8 (7.0)	15 (1.5)
*P*N_0_**	44 (57.9)	84 (52.5)	154 (51.9)	269 (59.7)	551 (54.4)
*P*N_1–3_	21 (27.6)	38 (23.8)	89 (29.1)	126 (27.9)	274 (27.8)
*P*N_4–10_	5 (6.6)	25 (15.6)	35 (11.8)	35 (7.8)	100 (10.2)
*P*N_>10_	6 (7.9)	13 (8.1)	19 (6.4)	21 (4.7)	59 (6.0)
Missing					28

*χ ^2^ = 2.18; *P* = .13.

**χ ^2^ = 3.06; *P* = .08.

**Table 4:** Human epidermal growth factor receptor 2 (HER2)-neu and labelling index according to age. Younger age correlates with HER2-neu positive status and high Ki67.

HER2 status					
	≤35 (%)	36–40 (%)	41–45 (%)	46–50(%)	Total (%)

HER2 0	14 (35.9)	31 (34.4)	52 (36.9)	100 (44.6)	197 (39.9)
HER2 1+	6 (15.4)	18 (20.0)	28 (19.9)	42 (18.8)	94 (19.0)
HER2 2+	6 (15.4)	16 (17.8)	28 (19.9)	45 (20.1)	95 (19.2)
HER2 3+	13 (33.3)	25 (27.8)	33 (23.4)	37 (16.5)	108 (21.9)
Missing					518

Labelling index—Ki 67					
Ki67 ≤10%	5 (8.1)	28 (23.1)	56 (24.2)	99 (27.3)	188 (24.2)
Ki67 >10%*	57 (91.9)	93 (76.9)	175 (75.8)	264 (72.7)	589 (75.8)
Missing					235

*χ ^2^ = 9.53; *P* = .002.

**Table 5:** Hormone receptor status stratified by age group. Younger age more frequently correlates with lack of hormone receptors.

HR status	PgR status			
	PgR+		PgR−	
	*N*	%	*N*

≤35 years				
ER+	43	58.1	6	8.1
ER−	6	8.1	19	25.7
Total	49	66.2	25	33.8

36–40 years					
ER+	106	66.3	18	11.3
ER−	5	3.1	31	19.4
Total	111	69.4	49	30.6

41–45 years				
ER+	199	66.1	26	8.6
ER−	15	5.0	61	20.3
Total	214	71.1	87	28.9

46–50 years				
ER+	328	72.6	34	7.5
ER−	19	4.2	71	15.7
Total	347	76.8	105	23.2

*χ* ^2^ = 7.07; *P* = .0078.

ER: estrogen receptor. HR: hormone receptor. PgR: progesterone receptor.

**Table 6:** Adjuvant treatment according to hormone receptor status and age. Endocrine therapy is more frequently administered in HR+ patients, independently of age (*χ* ^2^ = 699.89; 6 df; *P* < .0001).

		HT		CHT		CHT-HT	
		*N*	%	*N*	%	*N*	%

ER+/PgR+	≤35	0	0	3	7.1	39	92.9
	36–40	10	9.5	8	7.6	87	82.9
	41–45	40	20.4	6	3.1	150	76.5
	46–50	78	23.9	5	1.5	243	74.5

ER+/PgR−	≤35	0	0	0	0	6	100.0
	36–40	1	5.6	1	5.6	16	88.9
	41–45	3	12.0	2	8.0	20	80.0
	46–50	10	29.4	2	5.9	22	64.7

ER−/PgR+	≤35	0	0	2	33.3	4	66.7
	36–40	0	0	0	0	5	100.0
	41–45	0	0	5	33.3	10	66.7
	46–50	3	15.8	5	26.3	11	57.9

ER−/PgR−	≤35	0	0	17	89.5	2	10.5
	36–40	0	0	29	93.5	2	6.5
	41–45	1	1.7	55	91.7	4	6.7
	46–50	0	0	65	95.6	3	4.4

**Table 7:** Type of adjuvant therapy in the young population and in the subgroups stratified by age.

	≤35 (%)	36–40 (%)	41–45 (%)	46–50(%)	Total (%)
No therapy	1 (1.3)	1 (0.6)	5 (1.6)	6 (1.3)	13 (1.3)
HT alone	0	12 (7.4)	46 (14.9)	93 (20.1)	151 (14.9)
CHT alone	23 (29.5)	39 (23.9)	70 (22.7)	80 (17.3)	212 (20.9)
CHT followed by HT	54 (69.2)	111 (68.1)	187 (60.7)	284 (61.3)	636 (62.8)
